# Skipping the Biopsy: Real-World Experience of Whole-Exome Sequencing as First-Tier Testing in Pediatric Muscular Disorders

**DOI:** 10.3390/ijms27052446

**Published:** 2026-03-06

**Authors:** Chung-Lin Lee, Ya-Hui Chang, Chih-Kuang Chuang, Huei-Ching Chiu, Yuan-Rong Tu, Yun-Ting Lo, Jun-Yi Wu, Hsiang-Yu Lin, Shuan-Pei Lin

**Affiliations:** 1 Department of Pediatrics, MacKay Memorial Hospital, Taipei 104217, Taiwan; clampcage@gmail.com (C.-L.L.); wish1001026@gmail.com (Y.-H.C.); g880a01@mmh.org.tw (H.-C.C.); 2 Institute of Clinical Medicine, National Yang-Ming Chiao-Tung University, Taipei 112304, Taiwan; 3 International Rare Disease Center, MacKay Memorial Hospital, Taipei 104217, Taiwan; andy11tw.e347@mmh.org.tw (Y.-T.L.); wl01723138@gmail.com (J.-Y.W.); 4 Department of Medicine, Mackay Medical University, New Taipei City 252005, Taiwan; 5 Department of Nursing, Mackay Junior College of Medicine, Nursing and Management, New Taipei City 252005, Taiwan; 6 Division of Genetics and Metabolism, Department of Medical Research, MacKay Memorial Hospital, Taipei 104217, Taiwan; mmhcck@gmail.com (C.-K.C.); likemaruko@hotmail.com (Y.-R.T.); 7 College of Medicine, Fu-Jen Catholic University, New Taipei City 242062, Taiwan; 8 Department of Medical Research, China Medical University Hospital, Taichung 404327, Taiwan; 9 Department of Infant and Child Care, National Taipei University of Nursing and Health Sciences, Taipei 112303, Taiwan

**Keywords:** congenital myopathy, diagnostic yield, muscle biopsy, muscular dystrophy, whole-exome sequencing

## Abstract

Muscle biopsy has long been regarded as the cornerstone for diagnosing pediatric muscular disorders; however, it is invasive and may be limited by sampling error and inconclusive histopathological findings. This study aimed to evaluate whether whole-exome sequencing (WES) can effectively replace muscle biopsy as a first-line diagnostic approach in children with suspected neuromuscular disorders. Between January 2018 and December 2025, we prospectively enrolled 47 pediatric patients presenting with clinical features suggestive of muscular disorders at a tertiary medical center in Taiwan. The cohort included patients with suspected muscular dystrophies (*n* = 21), congenital myopathies (*n* = 23), and multiplex ligation-dependent probe amplification (MLPA)-negative Duchenne muscular dystrophy (DMD; *n* = 3). All patients underwent WES as the initial diagnostic test without prior muscle biopsy. Trio-based analysis using parental samples was performed in 29.8% of cases. Variant interpretation followed the American College of Medical Genetics and Genomics (ACMG) guidelines. WES identified a definitive molecular diagnosis in 72.3% of patients (34/47). Diagnostic yields varied by subgroup: 100% (3/3) in MLPA-negative DMD, 71.4% (15/21) in muscular dystrophies, and 69.6% (16/23) in congenital myopathies. Pathogenic or likely pathogenic variants were detected in 31 distinct genes, including COL6A1 and COL6A3, which are associated with Ullrich congenital muscular dystrophy. Notably, 58.8% of diagnosed patients (20/34) received molecular diagnoses that differed from their initial clinical impression, encompassing conditions such as ZSWIM6-associated neurodevelopmental disorders, GJB2-related hearing loss, OCRL-associated Lowe syndrome, and various metabolic or syndromic disorders. In all three MLPA-negative DMD cases, WES identified point mutations amenable to mutation-specific therapies. No patient required a muscle biopsy for diagnostic confirmation during the study period. First-tier WES demonstrates high diagnostic utility in pediatric muscular disorders while avoiding invasive muscle biopsy. The high rate of diagnostic reclassification underscores the substantial phenotypic overlap between primary neuromuscular diseases and other neurological or systemic conditions. These findings support the early implementation of genetic testing to enable accurate diagnosis and timely initiation of targeted therapies.

## 1. Introduction

Inherited neuromuscular disorders comprise a clinically and genetically heterogeneous group of conditions affecting skeletal muscle, the neuromuscular junction, peripheral nerves, or motor neurons [1,2]. Pediatric-onset muscular disorders, including muscular dystrophies and congenital myopathies, represent a major subset with significant clinical and psychosocial impact on affected children and their families. The combined prevalence of genetic muscle diseases has been estimated at approximately 1 in 3500 live births [3,4]. Common clinical manifestations include progressive muscle weakness, hypotonia, delayed motor development, and elevated serum creatine kinase levels. However, substantial phenotypic overlap among distinct disease entities often complicates accurate diagnosis [5,6].

For decades, muscle biopsy has been considered the diagnostic gold standard for pediatric muscular disorders [7,8]. Histopathological evaluation, supplemented by immunohistochemistry and electron microscopy, can reveal characteristic abnormalities such as dystrophic changes, protein deficiencies, and specific myopathic features, including nemaline rods, central cores, and fiber-type disproportion [9,10]. Nevertheless, muscle biopsy has notable limitations. As an invasive procedure frequently requiring general anesthesia in young children, it carries procedural risks such as bleeding, infection, wound complications, and anesthesia-related adverse events [11,12]. Furthermore, sampling error may occur if the biopsy site does not adequately reflect the underlying pathology, and histological findings may be nonspecific or normal in early disease stages or certain myopathy subtypes [13,14]. Previous studies have reported that up to 40% of muscle biopsies performed for suspected neuromuscular disorders fail to yield a definitive diagnosis [15].

The advent of next-generation sequencing (NGS) technologies has revolutionized the diagnostic approach to hereditary neuromuscular diseases [16,17]. Whole-exome sequencing (WES), which interrogates the protein-coding regions constituting approximately 1–2% of the human genome yet harboring nearly 85% of known disease-causing variants, has emerged as a powerful diagnostic tool [18,19]. Reported diagnostic yields of WES in pediatric neuromuscular cohorts range from 26% to 70%, depending on patient selection, prior testing, and analytical strategies [20,21,22,23,24]. Importantly, WES enables the identification of unexpected molecular diagnoses in patients whose clinical features resemble primary myopathies but are attributable to syndromic, metabolic, or neurodevelopmental disorders—a phenomenon known as diagnostic reclassification [25,26].

The “genetics-first” approach has gained increasing acceptance in pediatric neurology [27,28]. This paradigm advocates for early comprehensive genetic testing, potentially preceding or replacing invasive diagnostic procedures such as muscle biopsy. This strategy may shorten the diagnostic journey, reduce healthcare costs, minimize patient burden, and facilitate early access to mutation-specific therapies [29,30,31]. Early molecular diagnosis is particularly critical in Duchenne muscular dystrophy (DMD), where eligibility for exon-skipping therapies or nonsense mutation readthrough agents such as ataluren depends on the underlying genetic variant [32,33]. Similarly, precise genetic diagnosis in collagen VI-related myopathies and other congenital myopathies informs clinical surveillance, rehabilitation planning, and prognostic counseling [34,35].

Despite these advances, real-world data supporting the use of WES as a first-tier diagnostic test in Asian pediatric populations with suspected muscular disorders remains limited. Most published studies have employed WES after the failure of conventional diagnostic approaches rather than as an initial investigation [36,37]. The feasibility and effectiveness of a biopsy-sparing diagnostic strategy in routine clinical practice as well as its ability to identify phenocopies of primary muscle disease warrant further evaluation.

In this study, we present a 7-yr real-world experience using WES as the first-line diagnostic modality in 47 Taiwanese pediatric patients with suspected muscular dystrophies or congenital myopathies at a tertiary referral center. We aimed to assess the diagnostic yield of this genetics-first approach, delineate the spectrum of genetic findings, including unexpected diagnoses, and evaluate whether early implementation of WES can effectively obviate the need for muscle biopsy in clinical practice.

## 2. Results

### 2.1. Patient Characteristics

During the 7-yr study period (January 2018–December 2025), 161 pediatric patients presenting with clinical features suggestive of genetic myopathy were evaluated at our institution. Of these, 114 patients (70.8%) obtained definitive molecular diagnoses through conventional genetic testing methods. These included MLPA for *DMD* deletions or duplications (*n* = 60) and *SMN1* analysis for spinal muscular atrophy (*n* = 31), single-gene sequencing (*n* = 8), targeted gene panel testing (*n* = 10), and mitochondrial DNA sequencing (*n* = 2).

**Figure 1:**
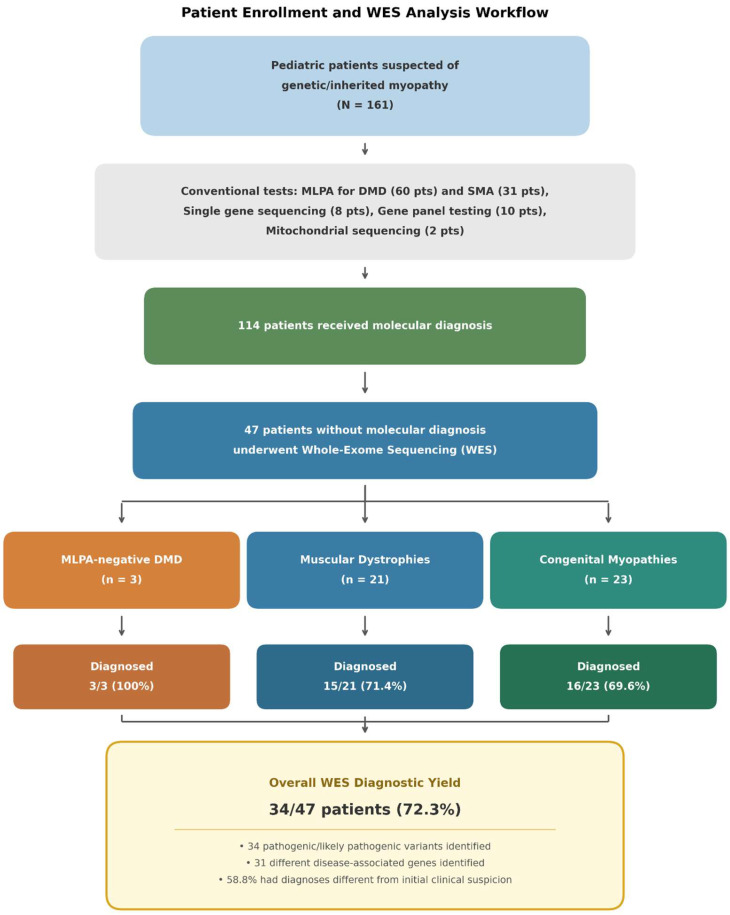
**Patient Enrollment and WES Analysis Workflow.** Flow diagram illustrating patient selection and diagnostic outcomes. Of 161 pediatric patients with suspected genetic myopathy, 114 received a molecular diagnosis through conventional testing (including MLPA for DMD and spinal muscular atrophy, single-gene sequencing, targeted gene panels, or mitochondrial DNA sequencing). The remaining 47 patients underwent WES. Patients were classified into three clinical subgroups: MLPA-negative DMD (*n* = 3), muscular dystrophies (*n* = 21), and congenital myopathies (*n* = 23). The overall diagnostic yield of WES was 72.3% (34/47), with subgroup-specific yields of 100% (3/3), 71.4% (15/21), and 69.6% (16/23), respectively. A total of 31 disease-associated genes were identified, and 58.8% of patients with a molecular diagnosis were reclassified relative to their initial clinical diagnosis. DMD, Duchenne muscular dystrophy; MLPA, multiplex ligation-dependent probe amplification; SMA, spinal muscular atrophy; WES, whole-exome sequencing.

The remaining 47 patients (29.2%) who did not achieve a molecular diagnosis using conventional approaches were subsequently enrolled for WES (Figure 1). Baseline demographic and clinical characteristics of these patients are summarized in Table 1. The cohort included 28 males (59.6%) and 19 females (40.4%). The median age at WES testing was 6.1 yr (range, 3.6–39.3 yr), with a mean ± standard deviation of 8.4 ± 8.2 yr. Based on initial clinical evaluation, patients were categorized into three diagnostic groups: MLPA-negative DMD (*n* = 3, 6.4%), MD (*n* = 21, 44.7%), and CM (*n* = 23, 48.9%). Trio-based WES was performed in 14 patients (29.8%) to facilitate the detection of *de novo* variants and improve variant interpretation accuracy.

### 2.2. Overall Diagnostic Yield

WES established a definitive molecular diagnosis in 34 of the 47 patients, resulting in an overall diagnostic yield of 72.3% (Table 2). All causative variants identified were classified as pathogenic or likely pathogenic according to the ACMG and Association for Molecular Pathology criteria. Thirteen patients (27.7%) remained without a molecular diagnosis despite comprehensive WES analysis; these cases are currently undergoing longitudinal follow-up with periodic reanalysis as new gene–disease associations are reported.

### 2.3. Diagnostic Yield by Clinical Subgroup

The diagnostic performance of WES varied across the three predefined clinical subgroups, as summarized in Table 2. The MLPA-negative DMD subgroup demonstrated the highest diagnostic yield. In these cases, WES identified pathogenic DMD point mutations, including nonsense variants and small insertions or deletions, which are not detectable by MLPA. This finding underscores the limitation of MLPA, which primarily detects large exon-level deletions or duplications accounting for approximately 65–70% of dystrophinopathy cases.

Among patients classified as having MD and CM, diagnostic yields were comparable (Table 2). The slightly lower diagnostic yield observed in CM likely reflects substantial phenotypic overlap with non-myopathic disorders, as well as technical limitations of WES in detecting certain variant classes, including deep intronic variants, complex structural rearrangements, and repeat expansions.

### 2.4. Spectrum of Genetic Findings

**Figure 2:**
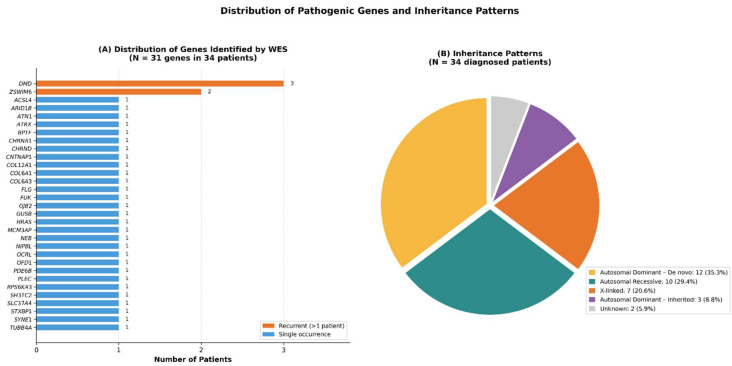
**Distribution of Pathogenic Genes and Inheritance Patterns Identified by Whole-Exome Sequencing.** (**A**) Horizontal bar chart showing the frequency of disease-associated genes identified in 34 molecularly diagnosed patients. Orange bars represent genes identified in more than one patient, whereas blue bars indicate genes identified in a single patient. *DMD* (*n* = 3) and *ZSWIM6* (*n* = 2) were the most frequently identified genes. (**B**) Autosomal dominant—de novo variants were most common (35.3%, *n* = 12), followed by autosomal recessive inheritance (29.4%, *n* = 10), X-linked inheritance (20.6%, *n* = 7), autosomal dominant—inherited variants (8.8%, *n* = 3), and unknown inheritance (5.9%, *n* = 2).

The 34 molecularly diagnosed cases harbored pathogenic or likely pathogenic variants across 31 distinct disease-associated genes, highlighting the pronounced genetic heterogeneity of pediatric neuromuscular disorders (Table 3 and Figure 2). The most frequently implicated gene was *DMD*, identified in three patients (8.8%), all belonging to the MLPA-negative DMD subgroup. *ZSWIM6* was the second most commonly identified gene, detected in two unrelated patients (5.9%). These patients initially presented with hypotonia and motor delay suggestive of primary myopathy but were ultimately diagnosed with *ZSWIM6*-associated neurodevelopmental disorder, characterized by movement abnormalities and autistic features.

The remaining 26 genes were each identified in a single patient, reflecting extensive allelic and locus heterogeneity. Variants affecting genes encoding key structural components of muscle fibers were detected in several cases, including *COL6A1* and *COL6A3* (collagen VI-related myopathies, including Ullrich congenital muscular dystrophy), *COL12A1* (Bethlem myopathy type 2), *PLEC* (limb girdle muscular dystrophy), *SYNE1* (Emery-Dreifuss muscular dystrophy), and *NEB* (nemaline myopathy).

In addition, pathogenic variants were identified in genes associated with syndromic disorders with prominent muscular involvement (*NIPBL, HRAS, BPTF, CHRNA1*), inborn errors of metabolism (*SLC37A4, GUSB, FUK*), and primary neurodevelopmental disorders (*STXBP1, ATRX, ATN1, TUBB4A*).

### 2.5. Inheritance Patterns

Analysis of inheritance patterns among the 34 patients with definitive molecular diagnoses revealed substantial genetic diversity (Figure 2). Autosomal dominant inheritance due to de novo variants was the most frequent pattern, observed in 12 patients (35.3%), as confirmed by trio-based WES or segregation analyses. Autosomal recessive inheritance accounted for 10 cases (29.4%), including glycogen storage disease type Ib, mucopolysaccharidosis type VII, nemaline myopathy, Ullrich congenital muscular dystrophy, and multiple pterygium syndrome.

X-linked inheritance was identified in seven patients (20.6%), comprising three cases of MLPA-negative DMD as well as ACSL4-related intellectual disability, ATRX syndrome, Lowe syndrome (*OCRL*), and Joubert syndrome (*OFD1*). Three additional patients (8.8%) demonstrated autosomal dominant inheritance without confirmed de novo status (*BPTF, HRAS,* and *NIPBL*). In two patients (5.9%), inheritance patterns could not be conclusively determined because of incomplete parental testing or complex segregation.

### 2.6. Diagnostic Reclassification

**Figure 3:**
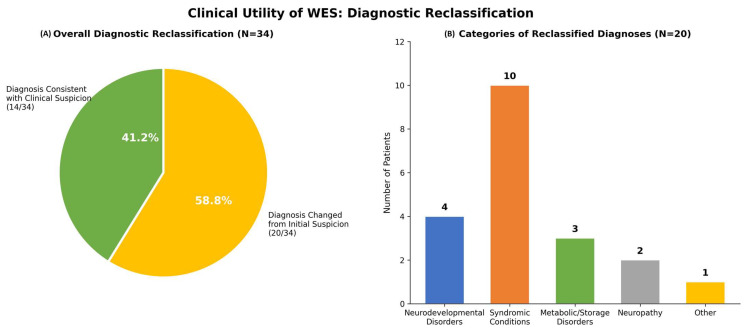
**Diagnostic Reclassification Following Whole-Exome Sequencing.** (**A**) Pie chart illustrating the proportion of patients whose final molecular diagnoses diverged from their initial clinical impressions. Among the 34 diagnosed patients, 58.8% (*n* = 20) were reclassified, whereas 41.2% (*n* = 14) had diagnoses consistent with clinical suspicion. (**B**) Bar chart depicting the distribution of reclassified diagnoses: syndromic disorders (*n* = 10), neurodevelopmental disorders (*n* = 4), metabolic or storage disorders (*n* = 3), hereditary neuropathies (*n* = 2), and other conditions (*n* = 1).

A major finding of this study was the high frequency of discordance between initial clinical impressions and final molecular diagnoses (Figure 3). Among the 34 patients who achieved a definitive molecular diagnosis, 20 (58.8%) were reclassified as having conditions outside the spectrum of primary myopathies or MD. In contrast, only 14 patients (41.2%) had molecular diagnoses concordant with the initial clinical suspicion.

Syndromic disorders constituted the largest category of reclassified diagnoses, accounting for 10 of 20 cases (50%). These included Coffin-Siris syndrome (*ARID1B*), Coffin-Lowry syndrome (*RPS6KA3*), Costello syndrome (*HRAS*), Noonan syndrome (*BPTF*), Escobar syndrome (*CHRNA1*), Lowe syndrome (*OCRL*), Joubert syndrome (*OFD1*), and ATRX syndrome. Many of these syndromes present with hypotonia and motor delay as early and prominent manifestations, which can obscure syndromic features during early childhood.

Neurodevelopmental disorders represented the second most frequent reclassification category (4/20, 20%), including developmental and epileptic encephalopathy type 4 (*STXBP1*), hypomyelinating leukodystrophy (*TUBB4A, MCM3AP*), and *ZSWIM6*-associated neurodevelopmental disorder. Metabolic and lysosomal storage disorders accounted for three cases (15%), including glycogen storage disease type Ib (*SLC37A4*), mucopolysaccharidosis type VII (*GUSB*), and a congenital disorder of glycosylation due to defective fucosylation (*FUK*). Two patients (10%) were diagnosed with hereditary neuropathies, including *SH3TC2*-related Charcot–Marie–Tooth disease and *CNTNAP1*-associated congenital hypomyelinating neuropathy. One additional patient received a diagnosis outside these major categories.

### 2.7. Clinical and Therapeutic Implications

The molecular diagnoses established through WES had direct and clinically meaningful implications for patient management. All three patients in the MLPA-negative DMD subgroup were confirmed to harbor pathogenic *DMD* point mutations. Precise mutation characterization is critical, as eligibility for mutation-specific therapies depends on the underlying variant. One patient carried a nonsense mutation potentially amenable to ataluren (Translarna), a readthrough therapy approved for ambulatory patients with nonsense mutation DMD. The mutation profiles of the remaining two patients provide essential information for evaluating eligibility for exon-skipping antisense oligonucleotide therapies.

For patients whose diagnoses were reclassified as non-myopathic disorders, molecular findings prompted substantial changes in clinical surveillance and treatment strategies. The patient diagnosed with mucopolysaccharidosis type VII due to biallelic *GUSB* variants was referred for evaluation for enzyme replacement therapy with vestronidase alfa. Patients with Costello and Noonan syndromes were enrolled in multidisciplinary follow-up programs addressing cardiac, oncologic, and developmental risks. Identification of collagen VI-related myopathies (*COL6A1*, *COL6A3*) led to the implementation of proactive respiratory surveillance because of the known risk of restrictive lung disease and nocturnal hypoventilation.

### 2.8. Avoidance of Muscle Biopsy

A notable outcome of this study was that no patient required a muscle biopsy for diagnostic confirmation throughout the study period. Among the 34 patients with definitive molecular diagnoses, genetic findings were sufficient to establish diagnoses without histopathological evaluation. In many cases, including syndromic, metabolic, and neurodevelopmental disorders, a muscle biopsy would not have provided diagnostic clarity, further supporting a genetics-first approach.

For the 13 patients who remained without molecular diagnoses, clinical management was guided by phenotypic assessment and supportive care. These patients continue to undergo longitudinal follow-up with periodic reanalysis of WES data as new gene-disease associations emerge. Overall, our experience demonstrates that a biopsy-sparing diagnostic strategy is both feasible and effective in the contemporary evaluation of pediatric patients with suspected neuromuscular disorders.

## 3. Discussion

This 7-yr prospective study demonstrates that WES is a highly effective first-line diagnostic tool for Taiwanese children with suspected muscular disorders. The overall diagnostic yield of 72.3% compares favorably with previously reported yields of 26–70% in pediatric neuromuscular cohorts, which vary depending on patient selection, prior genetic evaluation, and analytical strategies [20,21,22,23,24]. Importantly, WES was implemented as a true first-tier investigation without preceding muscle biopsy, distinguishing this study from earlier reports in which WES was applied only after extensive conventional testing [36,37]. These findings provide real-world evidence supporting a genetics-first diagnostic paradigm in pediatric neuromuscular practice.

### 3.1. Diagnostic Yield in Context

The diagnostic yield observed in our cohort aligns with—and in several subgroups exceeds—benchmarks reported by international centers. Tsang et al. [20] reported a 46% diagnostic yield using WES in a cohort of children with suspected genetic myopathy in Hong Kong, whereas Sanga et al. achieved a yield of 58% in an Indian cohort of patients with CM and MD [36]. Similarly, Kulsirichawaroj et al. [24] reported a 61% diagnostic rate using NGS in Thai pediatric patients with neuromuscular disorders unresolved by conventional diagnostic methods.

In comparison, the overall diagnostic yield of 72.3% in our cohort is notably higher. This enhanced yield likely reflects several contributory factors, including stringent patient selection criteria that excluded cases with clear non-genetic etiologies, the use of trio-based sequencing in nearly 30% of cases to facilitate the identification of de novo variants, and rigorous variant interpretation in accordance with current ACMG/AMP guidelines [38]. Moreover, the integration of detailed phenotypic assessment by experienced pediatric neurologists and clinical geneticists likely improved variant-to-phenotype correlation and diagnostic accuracy.

Subgroup analyses showed variation in diagnostic yield, although interpretation is limited by the small sample size in each category. The complete diagnostic success observed in the MLPA-negative DMD subgroup is consistent with the known mutational spectrum of dystrophinopathy: while MLPA efficiently detects large deletions and duplications accounting for approximately 65–70% of cases, the remaining cases are caused by point mutations, small insertions or deletions, and deep intronic variants that are readily captured by WES [39,40]. The somewhat lower yields in the MD (71.4%) and CM (69.6%) subgroups may reflect the greater genetic heterogeneity of these conditions, phenotypic overlap with non-myopathic disorders, and the inherent limitations of short-read exome sequencing in detecting structural rearrangements, repeat expansions, and deep intronic mutations [41,42].

### 3.2. Clinical Significance of Diagnostic Reclassification

One of the most clinically informative findings of this study was the high rate of diagnostic reclassification. Among the 34 patients who achieved a molecular diagnosis, 20 (58.8%) were ultimately diagnosed with conditions outside the spectrum of primary myopathies or MD. This phenomenon has been increasingly recognized in prior studies. Meyer et al. demonstrated that WES performed in a pediatric neuromuscular clinic frequently identifies both neuromuscular and neurodevelopmental disorders, with the latter constituting a substantial proportion of diagnoses [25]. Similarly, Piñeros-Fernandez et al. [26] emphasized the value of WES in uncovering diagnoses beyond initially suspected myopathic conditions.

The high reclassification rate observed in our cohort carries several important implications. First, it underscores the extensive phenotypic overlap between primary muscle diseases and syndromic, metabolic, and neurodevelopmental disorders, particularly in young children in whom the full clinical phenotype may not yet be evident [43,44]. Hypotonia and motor developmental delay—the most common referral indications to our neuromuscular clinic—are nonspecific features that may originate from pathology at any level of the neuraxis, ranging from the central nervous system to the muscle fiber itself [45]. Second, the diversity of reclassified diagnoses highlights the hypothesis-free nature of WES; unlike targeted gene panels, which are constrained by predefined disease categories, exome-wide analysis enables the identification of causative variants irrespective of the initial clinical suspicion [46,47]. Third, these findings suggest that rigid adherence to traditional diagnostic algorithms—wherein muscle biopsy precedes comprehensive genetic testing—may delay or preclude the identification of diagnoses outside the primary myopathy spectrum.

### 3.3. Therapeutic Implications and Precision Medicine

The molecular diagnoses established in this cohort had immediate and meaningful implications for clinical management. In the three patients with MLPA-negative DMD, precise characterization of the underlying point mutations was essential for determining eligibility for mutation-specific therapies. The therapeutic landscape for DMD has evolved substantially, with multiple exon-skipping antisense oligonucleotides approved or in advanced stages of development, as well as ataluren for ambulatory patients with nonsense mutations [32,33,48]. Consequently, early molecular diagnosis now directly informs treatment selection and enables access to disease-modifying therapies that may preserve ambulation and improve survival outcomes [49,50].

Beyond dystrophinopathies, diagnostic reclassification similarly prompted changes in clinical management. The patient diagnosed with mucopolysaccharidosis type VII became eligible for enzyme replacement therapy with vestronidase alfa, while patients with collagen VI-related myopathies were enrolled in structured respiratory surveillance programs due to their elevated risk of restrictive lung disease and nocturnal hypoventilation [34,51]. Patients diagnosed with RASopathies required multidisciplinary follow-up to address associated cardiac, oncologic, and developmental complications [52,53]. Collectively, these examples illustrate how accurate molecular diagnosis through WES extends beyond diagnostic confirmation to fundamentally reshape patient management and long-term care planning.

### 3.4. The Biopsy-Sparing Paradigm

A principal objective of this study was to evaluate whether first-line WES could obviate the need for muscle biopsy in pediatric patients with suspected muscular disorders. Our findings support this approach. Over the 7-yr study period, no patient required muscle biopsy for diagnostic confirmation. Among the 34 patients who achieved a molecular diagnosis, the genetic findings were considered definitive, and in many cases, the identified conditions—such as syndromic, metabolic, or neurodevelopmental disorders—would not have been detectable by muscle histopathology. For the 13 patients who remained molecularly undiagnosed, clinical management proceeded based on phenotypic evaluation and supportive care, with ongoing surveillance and periodic reanalysis of WES data as new gene–disease associations emerge.

This biopsy-sparing strategy offers several advantages. Muscle biopsy is an invasive procedure associated with procedural risks, including bleeding, infection, wound complications, and anesthetic adverse events, which are of particular concern in young children and patients with respiratory compromise [11,12]. Furthermore, the diagnostic yield of muscle biopsy is limited; up to 40% of biopsies in suspected neuromuscular disease have been reported to yield nonspecific or inconclusive results [15]. Sampling error, early disease stage, and subtype-specific histopathological subtlety further reduce diagnostic sensitivity [13,14]. In contrast, WES provides comprehensive germline analysis without tissue-specific limitations and has demonstrated favorable cost-effectiveness compared with traditional stepwise diagnostic pathways involving multiple sequential investigations [22,29,30].

Nevertheless, muscle biopsy retains a role in select clinical scenarios. In patients with clear myopathic features and persistently elevated CK levels in whom WES is uninformative, histopathological examination may reveal characteristic changes that guide further targeted genetic testing or suggest alternative diagnoses [7,8]. Certain conditions, including inflammatory myopathies and metabolic myopathies with distinctive ultrastructural features, may still require histological confirmation. Overall, however, our data support the use of upfront WES as the initial diagnostic investigation in most pediatric patients with suspected genetic myopathy, reserving muscle biopsy for carefully selected cases.

### 3.5. Genetic Heterogeneity and Emerging Disease Genes

The identification of pathogenic variants across 31 distinct genes in our cohort highlights the profound genetic heterogeneity of pediatric neuromuscular disorders. The most recent Gene Table of Neuromuscular Disorders lists over 600 genes associated with neuromuscular phenotypes, with new gene–disease associations reported at an accelerating pace [1]. This expanding genetic landscape presents challenges for targeted gene panels, which may become outdated and require frequent revision to maintain diagnostic sensitivity [16,17]. In contrast, WES captures the majority of protein-coding variants in a single assay and enables retrospective reanalysis as genomic knowledge evolves.

Several genes identified in this study merit particular attention. ZSWIM6, identified in two unrelated patients, has recently been linked to a neurodevelopmental disorder characterized by hypotonia, movement abnormalities, and autistic features [54]. Its detection in children initially suspected of having congenital myopathy illustrates the capacity of WES to uncover emerging disease entities that may be overlooked by conventional diagnostic strategies. Likewise, pathogenic variants in COL6A1 and COL6A3 confirmed diagnoses of collagen VI-related myopathies, encompassing Ullrich congenital muscular dystrophy and Bethlem myopathy—conditions with important prognostic and management implications, particularly regarding respiratory monitoring [34,55].

### 3.6. Limitations

Several limitations of this study should be acknowledged. First, the relatively small cohort size (*n* = 47), while sufficient to demonstrate feasibility and diagnostic utility, limits statistical power for subgroup comparisons. Second, as a single-center study conducted at a tertiary referral institution with established expertise in rare disease diagnostics, the generalizability of our findings to less specialized settings may be limited. Third, the inherent technical limitations of WES must be considered, as it does not reliably detect large structural variants, copy number changes beyond single-exon resolution, repeat expansions, deep intronic variants, or epigenetic alterations [41,42]. These variant classes likely account for a proportion of unsolved cases. Fourth, variants of uncertain significance were not systematically evaluated, and some may be reclassified as pathogenic with additional evidence over time.

Additionally, the absence of a parallel muscle biopsy cohort precludes direct comparison between genetic and histopathological diagnostic yields. However, the primary objective of this study was to assess the feasibility of a biopsy-sparing diagnostic strategy rather than to perform a head-to-head comparison. Despite this limitation, our findings clearly demonstrate that molecular diagnosis can be achieved without muscle biopsy in the majority of pediatric cases. Larger, multicenter studies with controlled designs are warranted to further validate this diagnostic paradigm.

### 3.7. Future Directions

For the 13 patients (27.7%) who remained molecularly undiagnosed, several investigative strategies may increase diagnostic yield. Periodic reanalysis of existing WES data as new gene–disease associations are identified is a cost-effective approach shown to improve diagnostic rates over time [56,57]. Whole-genome sequencing may identify pathogenic variants in noncoding regions and structural variants not captured by exome sequencing [58]. Long-read sequencing technologies offer improved detection of repeat expansions and complex rearrangements [59], while RNA sequencing from muscle tissue or fibroblasts can reveal splicing defects caused by deep intronic variants [60]. Functional studies, including transcriptomic, proteomic, and experimental modeling approaches, may further clarify the pathogenicity of unresolved variants. Finally, international data-sharing initiatives such as Matchmaker Exchange and ClinVar continue to facilitate the discovery of novel gene–disease associations in previously unsolved cases [61].

## 4. Conclusions

This study provides robust real-world evidence supporting the use of WES as a first-line diagnostic tool in Taiwanese pediatric patients presenting with clinical features suggestive of MD or CM. The diagnostic yield of 72.3%, achieved without the use of muscle biopsy, compares favorably with international benchmarks and supports the feasibility of a biopsy-sparing diagnostic approach. The high rate of diagnostic reclassification (58.8%) highlights the substantial phenotypic overlap between primary myopathies and syndromic, metabolic, and neurodevelopmental disorders, reinforcing the value of hypothesis-free genomic analysis. As mutation-targeted therapies continue to expand the therapeutic landscape of neuromuscular diseases, early and accurate molecular diagnosis is increasingly critical. Our findings support the broader adoption of genetics-first diagnostic algorithms in pediatric neuromuscular practice, with muscle biopsy reserved for selected cases in which genetic testing is inconclusive or histopathological confirmation is specifically indicated.

## 5. Materials and Methods

### 5.1. Study Design and Setting

This prospective observational study was conducted at MacKay Memorial Hospital, a tertiary referral medical center in Taipei, Taiwan, between January 2018 and December 2025. As a nationally designated center for rare disease management, the hospital receives referrals of pediatric patients with suspected hereditary neuromuscular disorders from across northern Taiwan. The primary objective of this study was to evaluate the diagnostic utility of WES as a first-line genetic investigation in children presenting with clinical features suggestive of muscular dystrophies or congenital myopathies, without prior muscle biopsy.

### 5.2. Participants

Patients were eligible for inclusion if they met the following criteria: (1) age < 18 yr at initial evaluation; (2) clinical features suggestive of a primary muscle disorder, including proximal or generalized muscle weakness, hypotonia, delayed motor development, elevated serum creatine kinase (CK) levels, or characteristic physical findings such as Gowers’ sign, calf pseudohypertrophy, or joint contractures; (3) absence of a definitive molecular diagnosis based on prior conventional genetic testing; and (4) provision of written informed consent by a legal guardian.

Patients were excluded if they had acquired causes of myopathy (e.g., inflammatory or drug-induced myopathies) or if clinical and laboratory findings were more consistent with motor neuron disease or peripheral neuropathy as the primary diagnosis.

Based on the initial clinical impression, patients were stratified into three subgroups: (1) MLPA-negative DMD, defined as patients with clinical features strongly suggestive of dystrophinopathy but negative for deletions or duplications on multiplex ligation-dependent probe amplification (MLPA) testing; (2) muscular dystrophies (MD), comprising patients with progressive muscle weakness and persistently elevated CK levels indicative of a dystrophic process; and (3) congenital myopathies (CM), including patients with early-onset hypotonia, delayed motor milestones, and relatively stable or slowly progressive weakness.

### 5.3. Clinical Evaluation

All patients underwent a comprehensive clinical evaluation by pediatric neurologists and clinical geneticists. Assessments included detailed medical histories focusing on age at symptom onset, motor developmental milestones, disease progression, and family history of neuromuscular disorders. Physical examinations documented the distribution and severity of muscle weakness using the Medical Research Council grading scale, presence of muscle atrophy or hypertrophy, deep tendon reflexes, joint range of motion, and associated clinical features such as facial weakness, ptosis, ophthalmoplegia, respiratory involvement, or cardiomyopathy.

Baseline laboratory investigations performed before genetic testing included serum CK, liver transaminases (aspartate aminotransferase and alanine aminotransferase), lactate dehydrogenase, and thyroid function tests. Electrodiagnostic studies, including nerve conduction studies and needle electromyography, were performed when clinically indicated to distinguish myopathic from neurogenic processes. Cardiac evaluations, including electrocardiography and echocardiography, were conducted in patients with suspected dystrophinopathy or when clinically warranted. Consistent with the genetics-first diagnostic strategy, muscle biopsy was not performed as part of the initial evaluation in any patient.

### 5.4. Whole-Exome Sequencing

Genomic DNA was extracted from peripheral blood leukocytes using standard procedures. WES was performed at accredited clinical laboratories, including Feng Chi Biotech Corp. (FCB; Taipei, Taiwan) and Taiwan Genomic Industry Alliance Inc. (TGIA; Taipei, Taiwan), using commercially available exome capture kits (Agilent SureSelect Human All Exon V6 or equivalent; Agilent Technologies, Santa Clara, CA, USA). Libraries were sequenced on Illumina platforms (HiSeq 2500 or NovaSeq 6000; Illumina Inc., San Diego, CA, USA) with paired-end 150 bp reads, targeting a minimum mean coverage of 100× across captured regions. Sequencing reads were aligned to the human reference genome (GRCh37/hg19 or GRCh38/hg38) using the Burrows–Wheeler Aligner (BWA-MEM v0.7.17), followed by variant calling using the Genome Analysis Toolkit (GATK v4.5.0.0) in accordance with best-practice guidelines.

Trio-based WES, involving sequencing of the proband and both biological parents, was performed in 14 patients (29.8%) to facilitate detection of de novo variants and accurate phasing of compound heterozygous variants. In the remaining cases, singleton WES was conducted, with targeted Sanger sequencing of parental samples performed subsequently to confirm variant segregation when candidate variants were identified.

### 5.5. Variant Interpretation and Classification

Variants were annotated using ANNOVAR (version 2020Jun08) and the Variant Effect Predictor (VEP, release 110). Candidate variants were filtered based on the following criteria: (1) minor allele frequency < 1% in population databases, including gnomAD (v4.0), the Taiwan Biobank, and the 1000 Genomes Project; (2) predicted functional consequence (missense, nonsense, frameshift, splice-site, or in-frame insertion/deletion); and (3) phenotypic relevance based on established gene–disease associations in Online Mendelian Inheritance in Man and the Human Gene Mutation Database.

Variant pathogenicity was evaluated according to the ACMG and Association for Molecular Pathology guidelines. In silico tools, including SIFT (v6.2.1), PolyPhen-2 (v2.2.3), MutationTaster 2021, and Combined Annotation Dependent Depletion (v1.7), were used to assess the potential impact of missense variants, while splice-site variants were analyzed using SpliceAI (v1.3.1) and Human Splicing Finder (v3.1). Variants were classified as pathogenic, likely pathogenic, variant of uncertain significance, likely benign, or benign. A definitive molecular diagnosis was assigned when a pathogenic or likely pathogenic variant consistent with the clinical phenotype and inheritance pattern was identified. Accordingly, cases were classified as ‘solved’ when a pathogenic or likely pathogenic variant consistent with the clinical phenotype was identified, or ‘unsolved’ when no definitive molecular diagnosis was reached.

### 5.6. Definition of Diagnostic Reclassification

Diagnostic reclassification was defined as the identification of a molecular diagnosis that differed substantially from the initial clinical impression. Specifically, cases were considered reclassified if WES revealed a disorder outside the spectrum of primary myopathies or MD, including syndromic conditions, neurodevelopmental disorders, metabolic diseases, or hereditary neuropathies. Cases were considered diagnostically concordant when the molecular findings confirmed a disorder within the initially suspected disease category, even if the specific genetic etiology had not been anticipated.

### 5.7. Statistical Analysis

Descriptive statistics were used to summarize demographic and clinical characteristics. Continuous variables are presented as mean ± standard deviation or median (range), as appropriate, and categorical variables are reported as frequencies and percentages. Diagnostic yield was defined as the proportion of patients receiving a definitive molecular diagnosis among those undergoing WES. Subgroup analyses were performed to compare diagnostic yields across the three clinical categories. All analyses were conducted using IBM SPSS Statistics version 26.0 (IBM Corp., Armonk, NY, USA).

### 5.8. Ethical Considerations

The study was conducted in accordance with the Declaration of Helsinki and was approved by the Institutional Review Board of MacKay Memorial Hospital (approval no. 21MMHIS109e; 1 October 2021). The research was performed under the protocol titled “The evaluation of genotype, phenotype, natural history, and cardiac function in patients with genetic rare diseases.” Written informed consent was obtained from all participants or their legal guardians prior to enrollment. For pediatric participants, parental consent and age-appropriate assent were obtained when applicable.

## Figures and Tables

**Table 1:** Baseline Characteristics of Patients Undergoing Whole-Exome Sequencing.

Characteristic	Value
Total patients, *n*	47
Sex, *n* (%)	
Male	28 (59.6)
Female	19 (40.4)
Age at WES (years)	
Median (range)	6.1 (3.6–39.3)
Mean ± SD	8.4 ± 8.2
Clinical diagnosis, *n* (%)	
MLPA-negative DMD	3 (6.4)
Muscular dystrophies	21 (44.7)
Congenital myopathies	23 (48.9)
Trio-based WES, *n* (%)	14 (29.8)

Abbreviations: DMD, Duchenne muscular dystrophy; MLPA, multiplex ligation-dependent probe amplification; SD, standard deviation; WES, whole exome sequencing.

**Table 2:** Diagnostic Yield of Whole-Exome Sequencing by Disease Group.

Disease Group	Total Patients	Diagnosed	Unsolved	Diagnostic Yield (%)
MLPA-negative DMD	3	3	0	100
Muscular Dystrophies	21	15	6	71.4
Congenital Myopathies	23	16	7	69.6
Total	47	34	13	72.3

Abbreviations: DMD, Duchenne muscular dystrophy; MLPA, multiplex ligation-dependent probe amplification.

**Table 3:** Molecular Diagnoses and Variant Details of Patients with Definitive Genetic Findings (N = 34).

No.	Group	Gene	Diagnosis	Inheritance	Age (yr)	Sex	Family Hx	Nucleotide Change	Protein Change	Zygosity	gnomAD MAF	TWB	ACMG
1	MLPA-negative DMD	*DMD*	Duchenne muscular dystrophy	X-linked (de novo)	5.1	M	No	c.7354G>T	p.Glu2452*	Hemizygous	Absent	Absent	Pathogenic
2	MLPA-negative DMD	*DMD*	Duchenne muscular dystrophy	X-linked (inherited)	3.9	M	Yes (maternal)	c.7993A>G	p.Asn2665Asp	Hemizygous	Absent	Absent	Likely pathogenic
3	MLPA-negative DMD	*DMD*	Duchenne muscular dystrophy	X-linked	9	M	No	c.5190G>C	p.Lys1730Asn	Hemizygous	Absent	Absent	Likely pathogenic
4	MD	*SLC37A4*	Glycogen storage disease type Ib	AR	1.6	M	No	c.1042_1043delCT/ c.898C>T	p.Leu348fs*53/p.Arg300Cys	Compound het	Absent	Absent	VUS/LP
5	MD	*PLEC*	Limb-girdle muscular dystrophy	AR	20	M	No	c.9343C>T/ c.13192G>A	p.Arg3115Cys/p.Ala4398Thr	Compound het	Absent	Absent	VUS/VUS
6	MD	*GJB2*	Bart-Pumphrey syndrome	AD (de novo)	17	M	No	c.109G>A	p.Val37Ile	Heterozygous	0.02	0.08	Pathogenic
7	MD	*SH3TC2*	Mononeuropathy of the median nerve	AD (de novo)	11	F	No	c.1817_1818del	p.Glu606Valfs*2	Heterozygous	Absent	Absent	LP
8	MD	*ARID1B*	Coffin-Siris syndrome	AD (de novo)	2.8	F	No	c.1717dup	p.Trp573Leufs*45	Heterozygous	Absent	Absent	Pathogenic
9	MD	*ACSL4*	X-linked intellectual disability	X-linked recessive	7.4	M	Yes (maternal)	c.1126-4T>C	Splice region	Hemizygous	Absent	Absent	VUS
10	MD	*MCM3AP*	Hypomyelinating leukodystrophy 5	AR	4.2	M	No	c.5383G>A/ c.998G>T	p.Ala1795Thr/ p.Gly333Val	Compound het	Absent	Absent	VUS/VUS
11	MD	*GUSB*	MPS VII	AR	9.6	F	No	c.104C>A/ c.1454C>T	p.Ala35Glu/ p.Ala485Val	Compound het	Absent	Absent	P/VUS
12	MD	*FUK*	CDG with defective fucosylation 2	AD (de novo)	3.1	M	No	c.428C>T/ c.1341+1G>T	p.Pro143Leu/ Splice donor	Compound het	Absent	Absent	LP
13	MD	*FLG*	Ichthyosis vulgaris	Unknown	6.7	M	N/A	c.3321delA	p.Ser1107Argfs*113	Heterozygous	0.005 (EA)	N/A	Pathogenic
14	MD	*STXBP1*	DEE4	AD (de novo)	2.1	F	No	c.1706C>G	p.Ser569Cys	Heterozygous	Absent	Absent	LP
15	MD	*PDE6B*	Retinitis pigmentosa	Unknown	15.4	M	N/A	c.610G>T	p.Glu204*	Heterozygous	Absent	Absent	Pathogenic
16	MD	*BPTF*	Noonan syndrome	AD	4.9	F	Yes (maternal)	c.205G>C	p.Gly69Arg	Heterozygous	Absent	Absent	LP
17	MD	*ZSWIM6*	NDD with movement abnormalities	AD (de novo)	3.8	M	No	c.481_482insT	p.Ala161fs	Heterozygous	Absent	Absent	LP
18	MD	*RPS6KA3*	Coffin-Lowry syndrome	AD (de novo)	7.1	F	No	c.2182C>T	p.Gln728*	Heterozygous	Absent	Absent	Pathogenic
19	CM	*ZSWIM6*	NDD with movement abnormalities	AD (de novo)	1.8	F	No	c.532_533insT	p.Ala178Valfs*76	Heterozygous	Absent	Absent	LP
20	CM	*OCRL*	Lowe syndrome	X-linked recessive	3.2	M	No	c.349+2T>G	Splice donor	Hemizygous	Absent	Absent	LP
21	CM	*ATRX*	ATRX syndrome	X-linked (de novo)	1.5	M	Yes (maternal carrier)	c.736C>T	p.Arg246Cys	Hemizygous	Absent	Absent	LP
22	CM	*HRAS*	Costello syndrome	AD (de novo)	2.9	M	No	c.34G>A	p.Gly12Ser	Heterozygous	Absent	Absent	Pathogenic
23	CM	*COL12A1*	Bethlem myopathy 2	AR	6.1	M	No	c.5894G>A	p.Gly1965Asp	Heterozygous	Absent	Absent	LP
24	CM	*SYNE1*	Emery-Dreifuss MD	AD (de novo)	8.2	M	No	c.5627G>A	p.Ser1876Asn	Heterozygous	Absent	Absent	LP
25	CM	*NIPBL*	Cornelia de Lange syndrome	AD	2.2	F	No	c.53G>C	p.Ser18Thr	Homozygous	Absent	Absent	LP
26	CM	*CNTNAP1*	Congenital hypomyelinating neuropathy	AD (de novo)	1.3	M	No	c.3361C>T	p.Arg1121*	Heterozygous	Absent	Absent	Pathogenic
27	CM	*TUBB4A*	Hypomyelinating leukodystrophy	AD (de novo)	8	F	No	c.1172G>A	p.Arg391His	Heterozygous	Absent	Absent	LP
28	CM	*OFD1*	Joubert syndrome	X-linked recessive	1.9	M	Yes (maternal)	c.2del	p.Met1fs	Hemizygous	Absent	Absent	LP
29	CM	*COL6A1*	Collagen VI myopathy	AD (de novo)	15	F	No	c.850G>A	p.Gly284Arg	Heterozygous	Absent	Absent	Pathogenic
30	CM	*ATN1*	Congenital hypotonia, epilepsy, DD	AD (de novo)	7	F	No	c.1449C>G	p.His483Gln	Heterozygous	Absent	Absent	LP
31	CM	*NEB*	Nemaline myopathy	AR	6.3	M	No	c.11606T>C/c.18800T>C	p.Ile3869Thr/p.Leu6267Pro	Compound het	Absent	Absent	LP
32	CM	*CHRND*	Multiple pterygium syndrome	AR	37	F	No	c.88C>T/c.107A>G	p.Arg30Trp/p.Lys36Arg	Compound het	Absent	Absent	VUS
33	CM	*CHRNA1*	Escobar syndrome	AD (de novo)	31	F	No	c.257G>A	p.Arg86His	Heterozygous	Absent	Absent	Pathogenic
34	CM	*COL6A3*	Ullrich CMD type 1	AR	17.5	M	No	c.6210+1G>A	Splice donor	Heterozygous	Absent	Absent	LP

Abbreviations: *, termination codon (stop codon); DEE4, developmental and epileptic encephalopathy 4; EA, East Asian; het, heterozygous; LP, likely pathogenic; MAF, minor allele frequency; MD, muscular dystrophy; MPS, mucopolysaccharidosis; NDD, neurodevelopmental disorder; P, pathogenic; TWB, Taiwan Biobank; VUS, variant of uncertain significance. “Absent” indicates the variant was not found in gnomAD v4.0 or Taiwan Biobank databases. For compound heterozygous cases, both variants are listed separated by “/”. Family history refers to relevant neuromuscular or genetic conditions in first-degree relatives.

## Data Availability

The datasets generated and analyzed during the current study are not publicly available due to patient privacy considerations and institutional review board restrictions. De-identified data may be made available by the corresponding author upon reasonable request and subject to appropriate ethical approval.
